# Single-Dose CpG Immunization Protects Against a Heterosubtypic Challenge and Generates Antigen-Specific Memory T Cells

**DOI:** 10.3389/fimmu.2015.00327

**Published:** 2015-06-25

**Authors:** Alexander J. Vogel, Deborah M. Brown

**Affiliations:** ^1^School of Biological Sciences, University of Nebraska-Lincoln, Lincoln, NE, USA; ^2^Nebraska Center for Virology, University of Nebraska-Lincoln, Lincoln, NE, USA

**Keywords:** immunization, CpG, influenza A virus, nasal mucosa, vaccines, cytotoxic T lymphocytes, CD4 T lymphocytes, CD8 T lymphocytes

## Abstract

Despite extensive research, influenza A virus (IAV) remains a major cause of morbidity, mortality, and healthcare expenditure. Emerging pandemics from highly pathogenic IAV strains, such as H5N1 and pandemic H1N1, highlight the need for universal, cross-protective vaccines. Current vaccine formulations generate strain-specific neutralizing antibodies primarily against the outer coat proteins, hemagglutinin and neuraminidase. In contrast to these highly mutable proteins, internal proteins of IAV are more conserved and are a favorable target for developing vaccines that induce strong T cell responses in addition to humoral immunity. Here, we found that intranasal administration with a single dose of CpG and inactivated x31 (H3N2) reduced viral titers and partially protected mice from a heterosubtypic challenge with a lethal dose of PR8 (H1N1). Early after immunization, vaccinated mice showed increased innate immune activation with high levels of MHCII and CD86 expression on dendritic cells in both draining lymph nodes and lungs. Three days after immunization, CD4 and CD8 cells in the lung upregulated CD69, suggesting that activated lymphocytes are present at the site of vaccine administration. The ensuing effector Th1 responses were capable of producing multiple cytokines and were present at least 30 days after immunization. Furthermore, functional memory responses were observed, as antigen-specific IFN-γ^+^ and GrB^+^ cells were detected early after lethal infection. Together, this work provides evidence for using pattern recognition receptor agonists as a mucosal vaccine platform for inducing robust T cell responses capable of protecting against heterologous IAV challenges.

## Introduction

Influenza A viruses (IAV) cause annual outbreaks of upper respiratory tract infection and induce severe infections in nearly three to five million people per year (1). In addition, IAV induces occasional pandemics, making it a prominent pathogen today. Influenza A virus outbreaks occur due to high rates of viral mutations in the outer coat proteins and reassortment of viral RNA segments stemming from antigenic drift (2) and antigenic shift (3), respectively. Due to these unpredictable events and poor predictive modeling, protection by current vaccines can be greatly reduced in some influenza seasons (4). For example, vaccine effectiveness has been shown to be 70–90% when circulating strains are well matched, but <50% if antigenic mutations occur (5). Influenza has increased deleterious effects on people at both ends of the age spectrum, with increased morbidity in the elderly and health complications in young children. Thus, the ability of the influenza virus to mutate and evade host immunity coupled with inadequate vaccines for at-risk populations highlights the need to develop a more universal influenza vaccine.

Current influenza vaccines are designed to generate humoral immunity against hemagglutinin (H) and neuraminidase (N) proteins in the vaccine strain. Master seed stalks are developed from either cold-adapted live virus or inactivated virus consisting of prevalent H1N1, H3N2, and one or two influenza B strains. Thus, the generation of high antibody titers and seroconversion to outer coat proteins in the vaccine strains are the main mechanisms of protection. However, antibodies induced by these vaccines have significant limitations if antigenic variation occurs. Therefore, universal vaccine approaches are currently being explored based on the fact that immunization with conserved influenza proteins elicits protection in many animal models following a lethal heterosubtypic challenge (6–8).

One approach to universal influenza vaccines is based on broadly neutralizing antibodies (9, 10). The majority of these studies focus on generating antibody responses to the stalk region of the HA protein that interfere with the ability of receptor-binding motifs to bind to sialic acid or inhibit fusion with the endosomal membrane, thus preventing infection. While broadly neutralizing antibodies can mediate sterilizing immunity, problems still persist with these approaches including low titers of stalk neutralizing antibodies after infection and/or overcoming the immune response to the immunodominant globular head of HA (11).

A second approach is to target T cells by vaccination. Successful universal IAV vaccines not only induce T cell responses with cytokine-producing and cytotoxic capabilities but also induce helper T cells responsible for generating optimal antibody responses (12) and maintaining CD8 memory (1). Furthermore, influenza-specific T cells not only respond to surface antigens but are also capable of mounting strong protective responses to much less variable regions of the influenza proteome including the nucleoprotein (NP), matrix (M), and polymerase proteins (13–15). Thus, T cell-based vaccines can be directed toward internal antigens that are well conserved between IAV subtypes and can circumvent outer coat mutations and viral escape variants to initiate protective immune responses.

As natural infection generates high levels of antigen-specific cells (16), CD8 cytotoxic T lymphocytes (CTL) responses have been established as a key cell type important for heterosubtypic infection. However, CD8 CTL is not the only cell type implicated in heterosubtypic protection. A role for CD4 cells is now becoming increasingly appreciated in establishing protection against heterologous IAV infections. Supporting observations for the importance of CD4 T cells include a role for cytolytic CD4 cells (17). These cells, via perforin dependent cytotoxicity in the absence of antibodies, have been shown to be important for protection against a lethal viral challenge (12, 18). Furthermore, vaccine platforms that induce T cell responses have translational applications as pre-existing memory T cells specific to internal influenza proteins have been associated with less virus shedding and lower symptom scores in humans (13, 19). Therefore, cell-mediated immunity should be able to provide a broad range of protection against serologically distinct viruses.

Consistently boosting and maintaining high levels of antigen-specific memory T cells still have not been achieved in humans as current influenza vaccines do not universally boost T cell responses across vaccinated individuals. One way to enhance vaccine efficacy and boost cellular responses is through the addition of adjuvants (20, 21). Unmethylated CpG, a TLR9 agonist, is currently being examined as an adjuvant in a number of clinical applications ranging from vaccine development to cancer immunotherapy (22). CpG is an attractive vaccine adjuvant not only for its potent immunostimulatory properties but also for its excellent stability, tolerability, and metabolism within a host.

Like IAV infection, CpG administration induces a Th1-biased response. Many of the immunological reactions that CpG induces result in adaptive responses that share a common mechanism with IAV infection. Indeed, both human and mouse studies have demonstrated that administration of CpG allows for enhanced T cell interferon-gamma (IFN-γ) production and CTL responses with antiviral capabilities *in vitro* and *in vivo*. (23–25). Because of the adjuvanticity in inducing robust T cell responses, CpG combined with inactivated influenza makes for a promising vaccine candidate. Here, we show that a single dose of CpG and inactivated influenza via the mucosal route promotes the development and differentiation of effector T cells that persist into memory and confer partial protection against a heterosubtypic influenza challenge. The current study highlights the evidence for the generation of a universal influenza vaccine that could not only provide protection against seasonal IAV variants but also highly virulent, potentially pandemic infections.

## Materials and Methods

### Mice

Male BALB/cByJ mice were purchased from Jackson Laboratories (RRID:IMSR_JAX:001026). Mice 6–8 weeks old were used in all experiments. Experimental animal procedures using mice were approved by and conducted in accordance with the Institutional Animal Care and Use Committee (IACUC) at the University of Nebraska-Lincoln.

### Mouse immunizations

For all immunizations, mice were under anesthesia using an isoflurane vaporizer. Mock immunized animals received 30 μl of PBS intranasally. For some groups, IAV was heat inactivated at 70°C for 1 h. One cohort of mice received 10 μl of inactivated virus containing 10^7^ EID_50_ A/HKx31-OvaII (x31/Ova), diluted in PBS for a total volume of 30 μl. A separate cohort of mice received 50 μg of CpG (ODN1826; Invivogen San Diego, CA, USA) combined with 10^7^ EID_50_ inactivated x31/Ova diluted in PBS. For x31/Ova priming, mice were anesthetized with isoflurane and infected with 950 EID_50_ x31/Ova virus also administered intranasally.

### Influenza virus challenge

For challenge experiments, mice were anesthetized with isoflurane, and A/Puerto Rico/8/1934 (PR8) was diluted in PBS and administered intranasally in a total volume of 30 μl. PR8 viruses were used at a sublethal dose of 0.1 LD_50_ or at a challenge dose of either 1 LD_50_ or 10 LD_50_. For challenge experiments, mice were infected with a lethal dose of PR8 4–6 weeks post immunization. Influenza viruses were generously provided by Dr. Paul Thomas, St. Jude Children’s Research Hospital (x31/Ova) or the Trudeau Institute (PR8).

### Extraction of RNA and real-time qRT-PCR

Mice were euthanized at various times following intranasal inoculation, lungs placed immediately in RNAlater (Ambion, Austin, TX, USA) and frozen at −20°C. The samples were weighed and homogenized in TRIzol (Ambion) at 1 ml/100 mg of lung tissue using a Tissue Tearor homogenizer (Biospec Products Inc., Bartlesville, OK, USA). RNA was isolated from lung homogenates, reverse-transcribed into cDNA, and amplified by quantitative real-time PCR (Step One Plus, Applied Biosystems) as previously described (26). Specific primers for murine TLR9 (Mm00446193_m1), IL-6 (Mm0044619_m1), TNF-α (Mm00443258_m1), MIP-1β (Mm00443111_m1) were purchased from Applied Biosystems. The following murine primer/probe sets were purchased from Integrated DNA Technologies (Coralville, IA, USA):

IFN-α_4_
5′-/56-FAM/TTTGGATTC/ZEN/CCCTTGGAGAAGGTGG/3IABKFQ/-3′ (probe),
5′-GCCTTCTGGATCTGTTGGTTA-3′ (forward)
5′-GCCTCACACTTATAACCTCGG-3′ (reverse)

CXCL10
5′-/56-FAM/ATCCCTCTC/ZEN/GCAAGGACGGTC/3IABKFQ/-3′ (probe),
5′-TGATTTCAAGCTTCCCTATGGC-3′ (forward),
5′-ATTTTCTGCCTCATCCTGCT-3′ (reverse)

To determine the viral titer, the following acid polymerase (PA) probe and primers were used:

5′-/56-FAM/CCAAGTCAT/ZEN/GAAGGAGAGGGAATACCGCT/3IABkFQ/-3′ (probe)
5′-CGGTCCAAATTCCTGCTGAT-3′ (forward),
5′-CATTGGGTTCCTTCCATCCA-3′ (reverse)

A known concentration of PA-containing plasmid was used to generate a standard curve in all reactions. PA copies per lung were then calculated based an initial concentration of 100 ng of cDNA as described (12, 26).

### Isolation of lung and lymph node cells for flow cytometry

Mice were euthanized at various times post infection, and lungs, draining lymph node (DLN) cells (a pool of mediastinal and cervical lymph nodes), or spleens were processed as described for flow cytometry analysis (26). Briefly, lungs were perfused with PBS, treated with collagenase D, and filtered through a 70-μm filter. DLN and spleens were dissociated into single cell suspensions and stained with fluorochrome-conjugated antibodies to CD4 (eBioscience Cat# 45-0042-80, RRID:AB_906231), CD8 (eBioscience Cat# 11-0081-82 RRID:AB_464915), CD49b (eBioscience Cat# 14-5971-85, RRID:AB_467767), TLR9 (eBioscience Cat# 11-9093-80 RRID:AB_465443), F4/80 (eBioscience Cat# 45-4801-80, RRID:AB_914344), CD103 (BioLegend Cat# 121405 RRID:AB_535948), CD69 (eBioscience Cat# 12-0691-82 RRID:AB_465732), CD11c (eBioscience Cat# 45-0114-82 RRID:AB_925727), CD11b (eBioscience Cat# 17-0112-81 RRID:AB_469342), I-A^d^ (BD Biosciences Cat# 553548 RRID:AB_394915), and CD86 (BD Biosciences Cat# 553692 RRID:AB_394994) for 30 min at 4°C. In some experiments, isolated lymphocytes were surface stained, fixed in 4% paraformaldehyde, and stained with anti-human GrB (Invitrogen Cat# MHGB05 RRID:AB_1500190) antibody to measure intracellular levels of GrB protein in effector T cells. Cells were acquired using a FACS Calibur (BD Biosciences) or Cytek DxP10 (Cytek Development, Fremont, CA, USA) flow cytometer and analyzed using FlowJo software (FlowJo, RRID:nif-0000-30575).

### Restimulation with peptides for cytokine analysis

For intracellular cytokine assays, cells were isolated from the lungs as described above and restimulated with IAV peptide pulsed A20s (ATCC, Manassas, VA, USA) as antigen-presenting cells (APCs) in RPMI 1640 containing 100 U/ml penicillin, 100 μg/ml streptomycin, 2 mM l-glutamine (Cellgro, Manassas, VA, USA), 7% FBS (Phenix Research Products, Candler, NC, USA), 10 mM HEPES (Fisher Scientific, Fair Lawn, NJ, USA), and 50 μM 2-ME (Sigma-Aldrich, St. Louis, MO, USA). Peptides used for *ex vivo* restimulation NP peptide 216–230 (RIAYERMCNILKGKF), NP peptide 146–159 (ATYQRTRALVRTGM), matrix peptides 164–179 (SHRQMVTTTNPLIRH), and matrix (M) peptide 211–226 (QARQMVQAMRTIGTH) were synthesized by New England Peptide (New England Peptide Inc., Gardner, MA, USA). Following restimulation for 2 h, Brefeldin A (Sigma-Aldrich) was added to T cell cultures at 10 μg/ml and maintained throughout the final 2–4 h of incubation. In some experiments, T cells were restimulated for 4–6 h, and Brefeldin A was added over night. After a total of 4–18 h in culture, T cells were surface stained with anti-CD4 and anti-CD8 antibodies as described above and fixed in 4% paraformaldehyde. Cells were then stained in saponin buffer (PBS containing 1% BSA, 0.1% NaN_3_, and 0.25% saponin) containing antibodies to IFN-γ (eBioscience Cat# 17-7311-82 RRID:AB_469504) and TNF-α (eBioscience Cat# 12-7321-81 RRID:AB_466198) for 40 min at room temperature in the dark. Cells were then washed and resuspended for FACS analysis. Cells were analyzed as described above.

### ELISA for detection of anti-influenza IgG2a antibodies

Ninety-six well plates were coated with x31/Ova virus (5 × 10^6^ EID_50_/ml) diluted in PBS overnight. Plates were washed with PBS and blocked for 1 h with PBS containing 2% FBS and 10 mM HEPES. Serum was added to the plates in blocking buffer and serially diluted twofold. After 2–3 h incubation at room temperature, alkaline phosphatase-conjugated goat anti-mouse IgG2a was added (Southern Biotech Associates, Birmingham, AL, USA) for 1 h at room temperature. Plates were developed using *p*-nitrophenyl phosphate (*p*-NPP) after a 15-min incubation in the dark. Absorbance was read at 405 nm and end point titers were calculated based on the dilution that gave two times the background optical density using serum from a naïve mouse as described (12).

### Statistics

Statistical significance between experimental groups was determined by one-way ANOVA followed by Tukey’s test using Prism 6.0 (Graph Pad Software).

## Results

**Figure 1:**
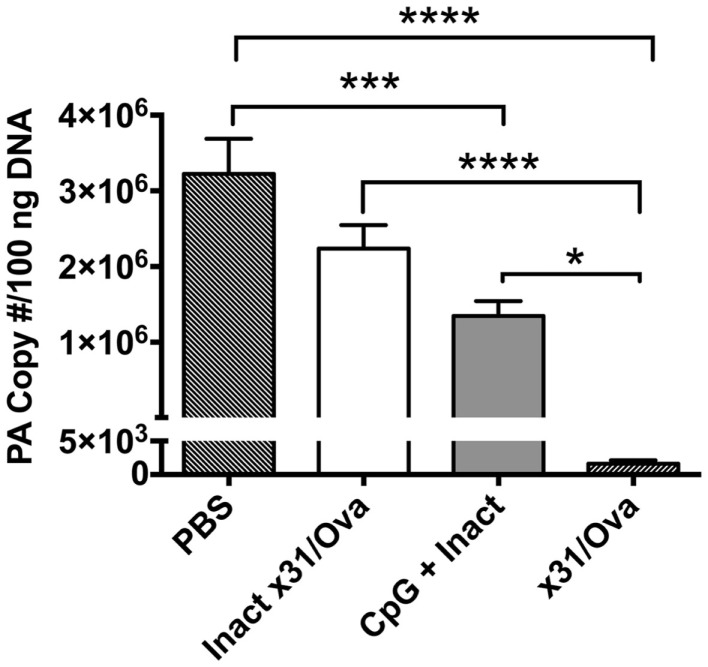
**CpG immunization lowers viral titers after a heterosubtypic influenza challenge**. BALB/cByJ mice were immunized intranasally and 4 weeks later challenged with 10LD_50_ of PR8. Seven days post challenge mice were euthanized and viral titers were determined via qRT-PCR. The amount of influenza RNA is shown as the PA copy number/100 ng of cDNA and is relative to a standard curve generated using known amounts of the IAV PA gene as described in the section “Materials and Methods.” Results are representative of two independent experiments (*n* = 4–5 mice per group), mean PA copy number + SEM. *p < 0.05, ***p < 0.001, ****p < 0.0001.

To demonstrate the *in vivo* protective efficacy of a single-dose intranasal vaccine using CpG and inactivated x31/Ova (CpG + Inact) mice were immunized and then challenged with a heterologous virus 4 weeks later. Subsequently, viral titers were measured 7 days post challenge. Viral burden in unimmunized groups were significantly higher than in CpG + Inact immunized mice (Figure 1). As expected, mice immunized with live x31/Ova had no appreciable levels of virus by day 7 (27). Sterilizing immunity was not observed with either immunization, as priming with H3N2 virus and challenging with H1N1 avoid contribution of antibody-mediated protection to H and N. Thus, the results suggest that a single immunization with CpG + Inact provides partial protection and may induce memory T cell responses capable of reducing viral titers and morbidity associated with a lethal heterosubtypic challenge.

**Figure 2:**
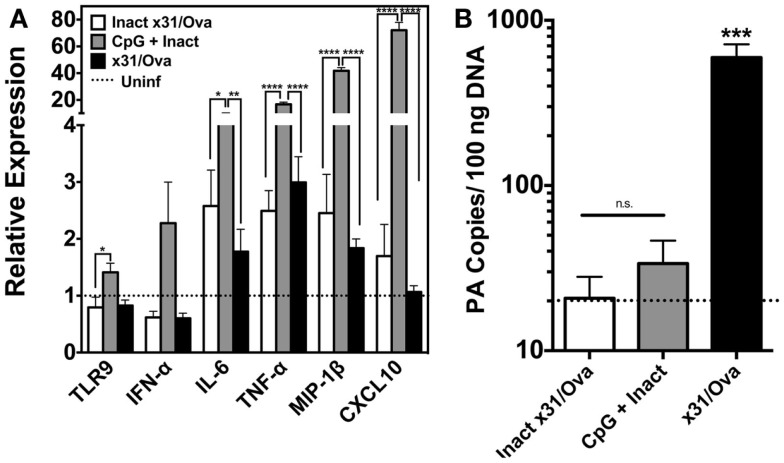
**CpG immunization induces the transcription of cytokine and inflammatory genes in the lung**. **(A)** BALB/cByJ mice immunized with the indicated regimens were euthanized 1 day after intranasal administration. RNA was extracted from whole lungs and qRT-PCR performed. Amount of mRNA transcripts for TLR9 and cytokines/chemokines is shown as arbitrary units relative to the amount of GAPDH mRNA present in each sample. Dashed line represents uninfected controls. **(B)** Influenza PA gene copy number was assessed in whole lungs 1 day after immunization as in Figure 1. Dashed line represents the limit of detection for the assay. Results represent the mean + SD of three to four mice per group *p < 0.05, **p < 0.01, ***p < 0.001, ****p < 0.0001.

To investigate the immunostimulatory capacity of this mucosally administered CpG-based vaccine, inflammatory cytokines, and chemokines were analyzed in the lung 1 day after intranasal administration. For inactivated influenza and x31/Ova cohorts, modest cytokine induction was observed characterized by IL-6 and tumor necrosis factor alpha (TNF-α) transcript upregulation. In contrast, immunization with CpG + Inact induced high levels of nearly all inflammatory and chemotactic transcripts tested (Figure 2A). TLR9 transcripts were also upregulated in the lung indicating a positive-feedback loop of cells capable of inducing inflammation. Next, the amount of viral PA copies present in the lung was determined 1 day after immunization. As shown in Figure 2B, only administration of live virus generated appreciable levels of the PA gene, confirming the attenuation of the vaccination strain.

Innate immune cells, including APC, play important roles in initiation and maintenance of appropriate immune responses to influenza infection (28–31). To determine whether innate immunity was modulated after immunization, a number of APC populations were examined in lymphoid tissues and the lung.

**Figure 3:**
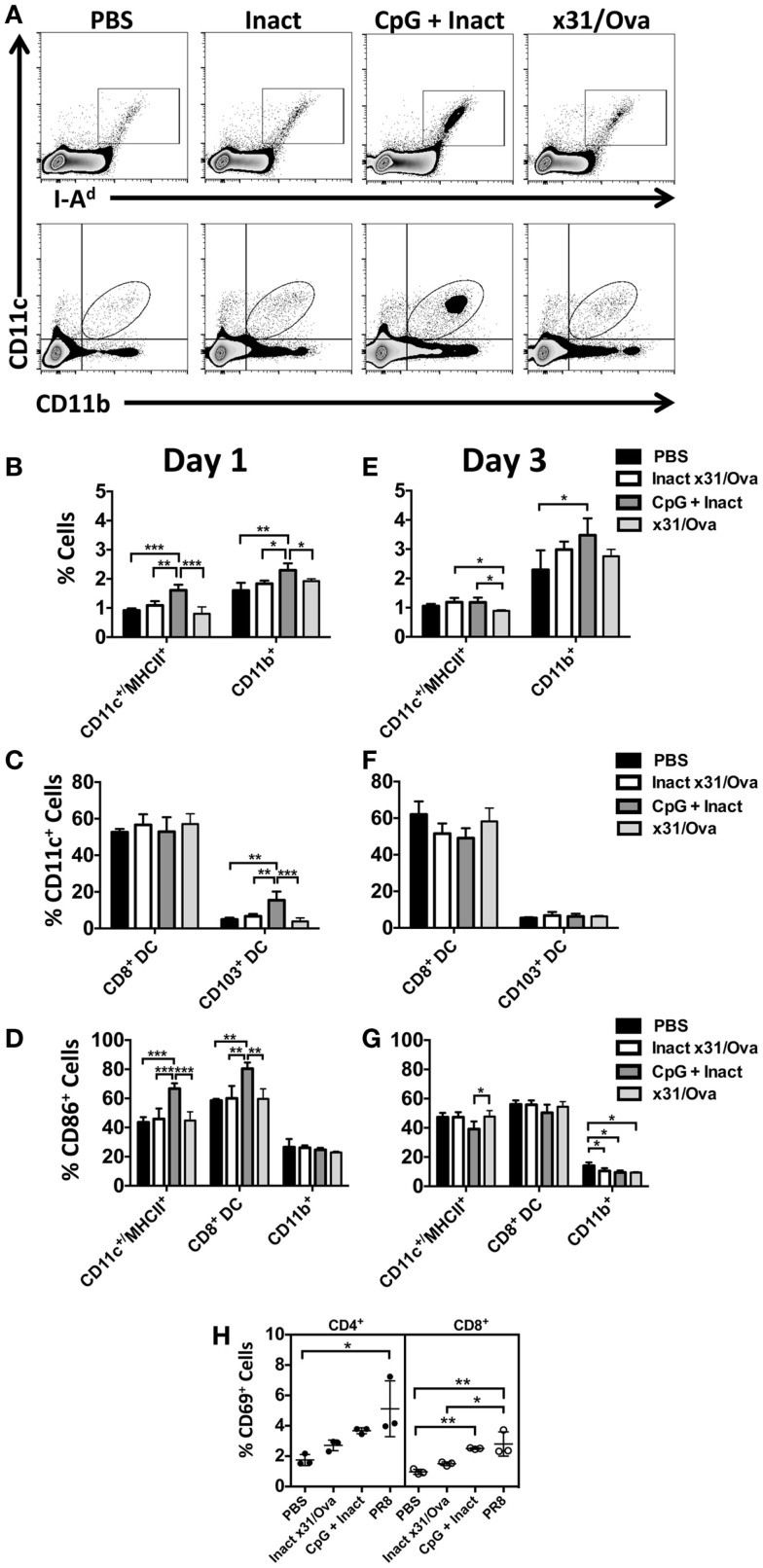
**CpG immunization induces APC maturation in the DLN**. BALB/cByJ mice were immunized intranasally. **(A)** Representative FACS plots from the DLN at day 1 post immunization. Innate immune populations were enumerated at day 1 **(B)** and day 3 **(E)** by flow cytometry. CD8^+^ DCs and CD103^+^ DCs were enumerated at day 1 **(C)** and day 3 **(F)**. Gated innate immune populations were assessed for their surface expression of the costimulatory molecule CD86 **(D,G)**. CD69 upregulation on the surface of CD4 and CD8 T cells in the draining lymph nodes 3 days after immunization **(H)**. Graphs represent two separate experiments (*n* = 3–5 mice per group) with data given in mean percentage ± SD. *p < 0.05 **p < 0.01, ***p < 0.001.

In contrast to x31/Ova infection, CpG + Inact immunization resulted in increased frequencies of innate immune cells in the DLN 1 day after immunization (Figure 3A). Rapid migration of CD11c^+^/MHCII^+^ cells as well as CD11b^+^ macrophages was observed in the DLNs (Figure 3B). Immunization also induced significant increases in the lung-resident CD103^+^ migratory DCs to the DLN; however, there were no detectable changes in the frequency of CD8^+^ DCs after any treatment (Figure 3C). To determine if vaccination induced APCs had the capacity to prime adaptive immune responses, expression of the costimulatory molecule CD86 was measured in the DLN populations. Upregulation of CD86 in CD11c^+^/MHCII^+^ and CD8^+^ DCs was observed, indicating that these cells may initiate naïve T cell responses (Figure 3D).

The results here are consistent with other published studies suggesting that the intranasal administration of CpG induces APC maturation in the DLNs (32). In contrast to the diverse influx of cells seen in the DLN at day 1, only the frequency of CD11b^+^ cells was increased after CpG immunization (Figures 3E,F). By day 3, minimal changes were observed in CD86 expression (Figure 3G). To determine the functional consequences of APC maturation, CD69 (a surface marker typically upregulated after T cell activation) expression on T cells was measured at day 3 in the lymph node (33, 34). Here, PR8 infection was used as a positive control. In the DLNs, CD69 expression was differentially upregulated in CD8 cells after CpG + Inact or influenza immunization while only PR8 infection resulted in increases in CD4 cells (Figure 3H). Interestingly, the mature APC phenotype observed in the CD8^+^ DC subset did correspond with the activation status of the CD8 cells (Figure 3H). These results suggest that CpG + Inact immunization induces a population of activated cells derived from naïve precursor cells in the DLN.

**Figure 4:**
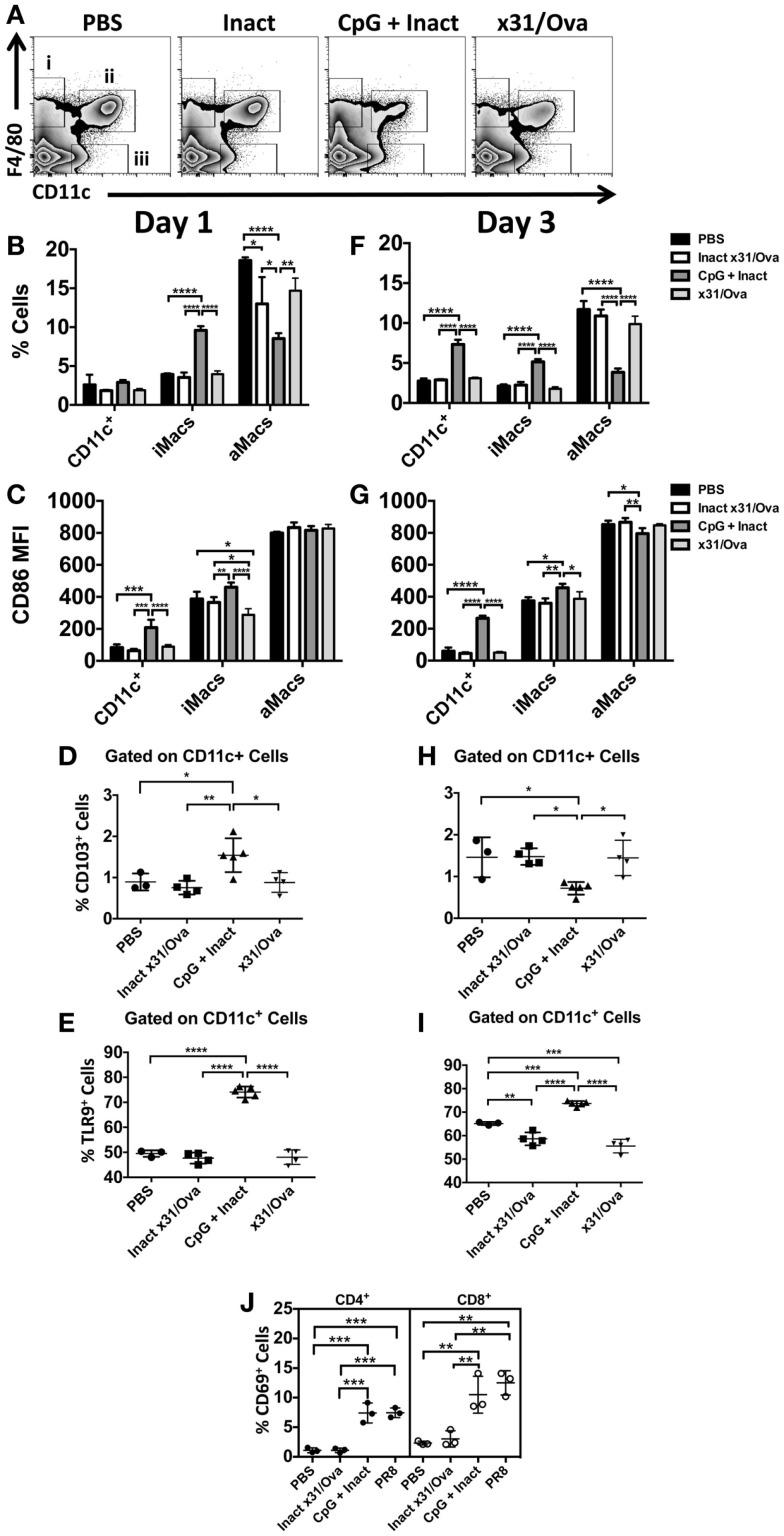
**CpG immunization induces APC maturation in the lung**. BALB/cByJ mice were immunized intranasally. **(A)** Representative FACS plots from the lung at day 1 show (i) interstitial macrophages (F4/80^+^), (ii) alveolar macrophages (F4/80^+^ CD11c^+^), and (iii) conventional DCs (CD11c^+^). DCs, interstitial macrophages, and alveolar macrophage populations were enumerated at day 1 **(B)** and day 3 **(F)** in the lungs. Innate immune cell populations were assessed for their surface expression of the costimulatory molecule CD86 by measuring the median fluorescence intensity (MFI) **(C,G)**. The influx of CD103^+^ dendritic cells **(D,H)** and TLR9^+^ **(E,I)** cells was also enumerated after immunization. CD69 upregulation on CD4 and CD8 T cells in the lungs 3 days after immunization **(J)**. Graphs represent two separate experiments (*n* = 3–5 mice per group) with data given in mean percentage ± SD. *p < 0.05, **p < 0.01, ***p < 0.001, ****p < 0.0001.

In contrast to the DLN, APC influx and activation were sustained in the lung until at least day 3. In the lung, we have identified three populations of cells: (i) interstitial macrophages (ii) alveolar macrophages, and (iii) conventional CD11c^+^ cells each with unique responses to CpG immunization (Figure 4A). While increases in the frequency of CD11c^+^ cells were not observed until day 3, interstitial macrophage influx was significantly higher at both time points in CpG + Inact immunized groups (Figures 4B,F). CpG + Inact treatment reduced the percentage of alveolar macrophages (F4/80^+^/CD11c^+^) in the lung similar to what has been reported for PR8 infection (35). Furthermore, we observed clear increases in the percentage of CD86^+^ cells (data not shown) as well as CD86 median fluorescence intensity (MFI) in CD11c^+^ and interstitial macrophages at day 1 and day 3 (Figures 4C,G). Interestingly, infection with x31/Ova demonstrated relatively low CD86 MFI levels, a similar profile to that of inactivated virus. CD103^+^ migratory dendritic cell populations were also examined in the lung. At day 1, increases in CD103^+^ DCs were observed (Figure 4D); however, these cells were decreased in frequency by day 3 (Figure 4H). Next, we wanted to determine the frequency of cells expressing the receptor for CpG (TLR9) in the lung. Significant increases in TLR9-expressing CD11c^+^ dendritic cells were observed over the time course compared to both inactivated and live virus immunizations (Figures 4E,I). Lastly, CD69 expression on T cells was examined in the lung 3 days after immunization. Compared with PBS and inactivated virus, both CpG + Inact and PR8 immunization induced significant increases in CD69^+^ T cells. We conclude that immunization with CpG and inactivated virus leads to a phenotypic maturation of APCs where favorable conditions exist for and early T cell activation in the DLN (Figure 3) and lung (Figure 4).

**Figure 5:**
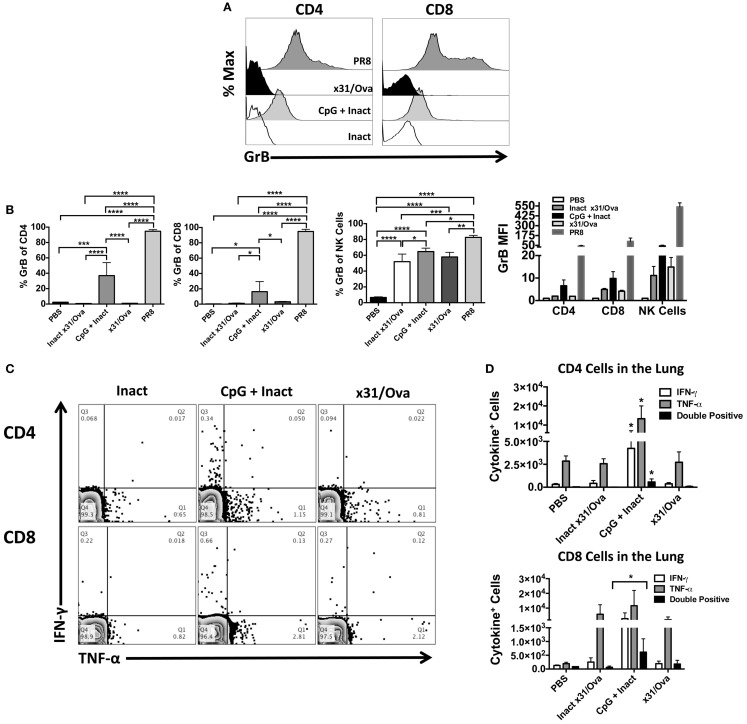
**CpG immunization and IAV infection induce effector T cell responses in the lung**. BALB/cByJ mice were immunized intranasally, mice were euthanized, and lungs harvested at day 6. **(A)** Representative overlay histograms show GrB expression after gating on CD4^+^, CD8^+^ T cells, or NK cells in the lung. **(B)** Shown are the percentages of CD4, CD8, or NK cells expressing GrB as well as the GrB MFI of each population. **(C)** Representative FACS plots in the lung after *ex vivo* restimulation for 4–6 h with NP and M peptides. **(D)** Absolute number of cytokine-producing CD4 and CD8 cells were enumerated after restimulation. Single * in the CD4 quantification denotes significance over all other groups as analyzed by ANOVA. Data represent two independent experiments (*n* = 4–5 mice per group) given in mean percentage of cytokine positive cells + SD *p < 0.05, **p < 0.01, ***p < 0.001, ****p < 0.0001.

Cytotoxic T cells have been shown to be important effectors in IAV infections in heterosubtypic protection in both humans (19) and mice (16). Thus, we sought to determine if T cells with cytolytic potential could be generated after vaccination. Six days after immunization, mice were euthanized and Granzyme B (GrB) expression in T cells was measured. As a positive control for GrB expression, infection with a sublethal dose of PR8 was used and induced >97% GrB^+^ CD4 and CD8 cells as expected (26, 36). Interestingly, compared to the x31/Ova infection, CpG + Inact immunized mice had a much higher percentage of GrB positive cells (Figure 5A). While the percentage of CD4 and CD8 T cells expressing GrB in inactivated or x31/Ova cohorts was <5%, immunization with CpG + Inact resulted in 40% GrB^+^ CD4 cells and 20% GrB^+^ CD8 cells (Figure 5B). Similarly, the GrB MFI was highest in CpG- and PR8-immunized mice. As expected, the CD8 MFI was universally higher than the CD4 GrB MFI. These results suggest that while inactivated influenza weakly induces T cell responses in the lung, addition of CpG enhances the effector profile and induces T cells with cytotoxic capabilities.

To further characterize the effector T cell response after immunization, cytokine production in the lung was assessed by intracellular staining. Previously, we have shown that restimulation with a peptide cocktail consisting of NP epitopes induces strong T cell cytokine production during the influenza response (26) and allows us to assess cross-reactive T cells after vaccination. At day 6, most cytokine-producing T cells were TNF-α^+^ while low frequencies of IFN-γ^+^ cells were observed after peptide restimulation (Figure 5C). A small cohort of these cells was dual IFN-γ and TNF-α producing cells in CpG + Inact and x31/Ova-primed groups (Figure 5D). Collectively, these results demonstrate that effector CD4 and CD8 cells were recruited to the lung during immunization and acute infection, and that antigen-specific responses were generated by day 6. Furthermore, these data suggest that different priming events occur between a natural infection and CpG + Inact immunization, which lead to differences in quality and quantity of effector T cells.

**Figure 6:**
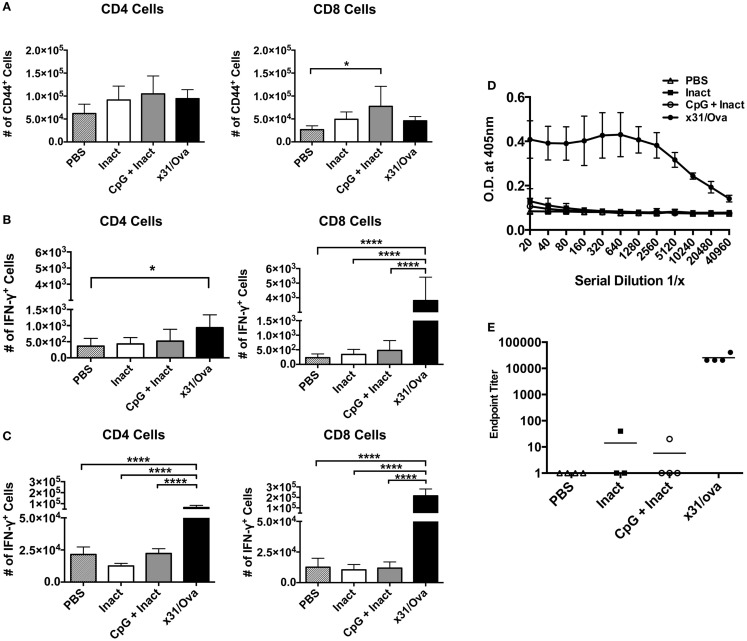
**Low-dose influenza infection generates antigen-specific memory cells in the spleen and lung**. BALB/cByJ mice were vaccinated with the indicated regiments and euthanized 4 weeks later. **(A)** Absolute numbers of CD44^+^ T cells were quantified in the lungs. Intracellular cytokine staining on cells isolated from the **(B)** lung or **(C)** spleen was performed after an 18-h incubation with NP and M peptides. Shown is the number of IFN-γ positive cells. Data represent two independent experiments (*n* = 4–5 mice per group) with data given in mean percentage of CD44^+^ or cytokine positive cells + SD. *p < 0.05, ****p < 0.0001. **(D)** Five weeks post immunization, serum was collected and serially diluted and the optical density at 405 nm was determined. **(E)** Endpoint titers for the anti-x31/ova IgG2a from samples in **(D)**. Data are given in mean ± SD. *p < 0.05, ****p < 0.0001.

Next, we sought to determine if the effector cells identified in the lung persisted into memory. Expression of CD44 has been shown as a memory T cell marker and is present on the surface of resting memory cells (37). Thus, 28 days after immunization, CD44^+^ cells were enumerated in the lungs and spleens. Although no differences in CD44 expression were observed in splenocytes (data not shown), CpG + Inact induced significant increases in the number of CD8^+^/CD44^+^ T cells in the lungs (Figure 6A). Next, *ex vivo* cytokine analysis was performed on cells isolated from the lungs and spleens. Unexpectedly, only increases in antigen-specific responses were detected in x31/Ova-immunized groups (Figures 6B,C). Therefore, CpG + Inact immunization induced CD44^+^ cells in the lungs; however, in the absence of a secondary challenge, cytokine responses were only detected in x31/Ova-infected animals. To confirm that the protection generated by our vaccine was in the absence of pre-existing antibodies, serum was collected 5 weeks after immunization and anti-x31/Ova IgG2a titers were measured. Antibody responses to the virus were only generated by prior infection (Figure 6D). Indeed, endpoint titers were very low after CpG + Inact immunization, and only one out of four mice generated any anti-influenza antibody response (Figure 6E). Thus, the protection mediated by CpG + Inact immunization does not depend on IgG2a antibodies.

**Figure 7:**
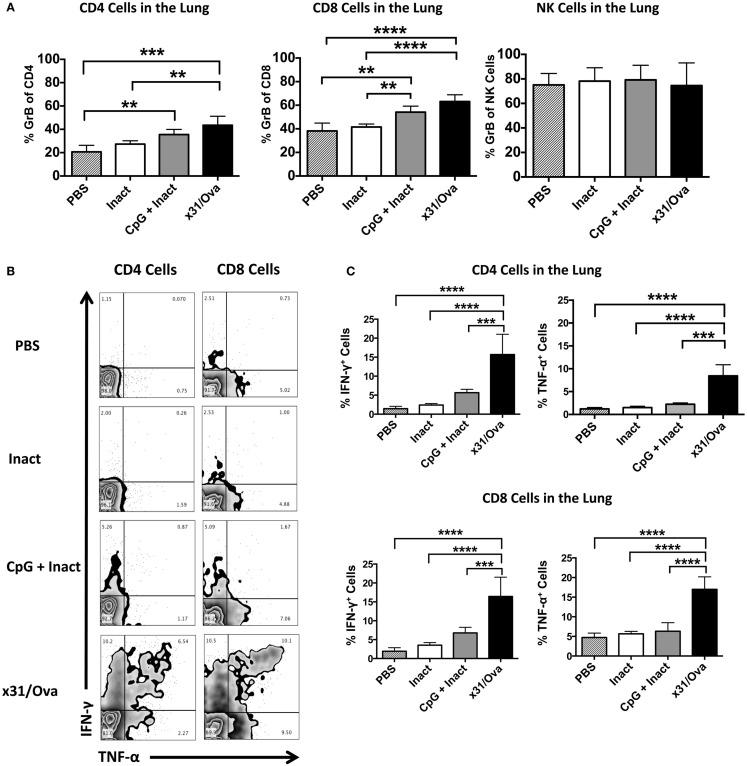
**CpG immunization induces functional memory responses at day 5 post challenge with a heterosubtypic IAV infection**. BALB/cByJ mice were vaccinated as indicated. Four to five weeks post immunization, mice were challenged with a 10 LD_50_ of PR8. **(A)** Lungs were harvested 5 days after infection, and the absolute number of GrB^+^ T and NK cells were enumerated via flow cytometry. **(B)** Representative FACS plots in the lung after *ex vivo* restimulation for 4–6 h with NP and M peptides. **(C)** Percentage of cytokine-producing CD4 and CD8 cells were enumerated after restimulation. Data represent two independent experiments (*n* = 4–5 mice per group) given in mean percentage of cytokine positive cells or absolute number of GrB^+^ cells + SD. *p < 0.05, **p < 0.01, ***p < 0.001, ****p < 0.0001.

Based on the requirement for secondary effector T cell responses to mediate protection in highly pathogenic infections (38), it was important to determine the frequency of GrB^+^ and antigen-specific cytokine-producing cells after influenza challenge. Mice were immunized and 4–5 weeks later challenged with a lethal dose of PR8. Five days after challenge, lungs were harvested and cells were analyzed for GrB expression. In contrast to mock or inactivated immunizations, administration of CpG + Inact or x31/Ova significantly enhanced the number of GrB^+^ T cells within the lung (Figure 7A). To further quantify memory T cell responses after IAV challenge, we sought to determine the number of cytokine expressing cells at the site of infection. In mock- and inactivated-immunized mice, the frequency of cytokine positive cells was low in both CD4 and CD8 cells at day 5 post challenge indicative of the lack of a primed T cell response (Figure 7B). In CpG + Inact immunized mice, T cell responses were apparent, characterized by IFN-γ and TNF-α-positive cells (Figure 7B). Furthermore, a robust memory response was initiated with high frequencies of dual cytokine positive T cells after x31/Ova immunization and PR8 challenge (Figure 7B). CpG + Inact immunization produced increased trends of cytokine responses compared to PBS groups; however, significance was not achieved (Figure 7C). These results suggest the presence of a naïve T cell response after challenge in mock or inactivated groups, highlighted by the lack of robust T cell responses at day 5. In contrast, the recall of memory T cells in CpG + Inact immunized or x31/Ova-infected animals was seen by the increased frequencies of GrB and cytokine positive T cells early after influenza challenge.

## Discussion

The use of pattern recognition receptor (PRR) agonists is becoming increasingly widespread as potential vaccine adjuvants for a number of diseases including IAV. Here, we describe a novel influenza vaccine platform based on the generation of antigen-specific CD4 and CD8 T cells capable of reducing viral titers after lethal IAV challenge. Single dose, intranasal administration of CpG + Inact resulted in the production of proinflammatory cytokines, mobilization of APC populations, and the establishment of effector T cell responses by day 6 post administration. Our results suggest that effector T cells transitioned into memory cells capable of rapidly responding in the lung upon reinfection to reduce viral replication in an antigen-specific manner.

The vaccine platform described here has numerous advantages compared to other IAV vaccines. First, the use of the PRR agonist, CpG, as an innate immune modulator has been approved for use in humans, but is not yet incorporated into current IAV vaccine preparations. In contrast to vaccines administered via the intradermal, subcutaneous, or intramuscular route, intranasal immunization induces immunity at mucosal surfaces where an effective local immune response is required following IAV infection. Additionally, this vaccine induces memory T cell responses in the lung capable of responding to internal conserved viral epitopes, which are thought to greatly aid in protection against heterosubtypic IAV challenges. Lastly, a single dose administered without boosting is a clear advantage for vaccine compliance in human populations.

Pattern recognition receptor agonists, especially CpG, can protect against infectious agents in two ways. One is non-specific immune activation involving the modulation of macrophages, NK cells, inflammatory cytokines, and polyreactive IgM molecules. Numerous studies have demonstrated the protective activation of innate immunity by exposing mice to a single dose of CpG against a number of pathogens including Ebola, anthrax, and malaria (39), which can induce non-specific protection lasting between 1 and 3 weeks. Additionally, we have observed protection against weight loss after IAV infection in an antigen-independent manner using CpG (Brown and Swain, unpublished). Many studies that examine the efficacy of CpG-based vaccines report survival, weight loss, and viral replication of IAV 1–3 weeks after the final boost or immunization (40–44). Even though in the majority of these studies antigen-specific T cell responses are quantified, the experiments performed under these conditions do not allow sufficient time after immunization for the non-specific effects of CpG immunization to wane. In contrast, our study allows the development of T cell memory responses (Figures 5 and 6) that participate in partial inhibition of viral replication (Figure 1).

Following an infection with a homotypic virus, strain-specific humoral immunity is induced and prevents reinfection upon subsequent challenges. To date, IAV vaccines use this approach to generate strain-specific antibodies against outer coat proteins in the common seasonal influenza strains. In a heterotypic influenza challenge model, T cells reactive to conserved viral proteins, such as NP and M, are capable of providing protection and persist in the tissues and secondary lymphoid organs as a source of memory cells to respond to serologically distinct IAV infections (19). However, it is unlikely that cross-reactive CTLs provide sterilizing immunity, as some degree of antigen processing and presentation must occur to initiate a memory response (45). Here, effector functions provided by CTLs (Figure 7) may have substantial impacts on viral replication (Figure 1) and therefore morbidity and mortality. Similar to infection with x31/Ova, vaccination with CpG + Inact does not provide sterilizing immunity after heterosubtypic challenge but reduces viral burden. Previous work from our lab suggests that reducing viral replication is one of the determinants for a positive survival outcome (26). Therefore, the protection provided by CD4 and CD8 T cells generated by vaccination is significant to the outcome of a heterotypic challenge.

Other factors likely contributed to the lack of total viral clearance by day 7. Upon challenge, a considerable population of dual cytokine-positive T cells was present in x31/Ova-immunized mice but not CpG + Inact-immunized mice. This suggests differences in priming between the two cohorts results in differences in quality of protection. Surprisingly, little innate immune modulation was observed in x31/Ova mice compared to CpG + Inact mice (Figures 2–4), suggesting that while inducing inflammation and APC modulation is important in a vaccine setting, low levels of viral replication may be the best strategy to induce robust T cell memory responses (46). Alternatively, T cell responses generated by CpG vaccination could be below a certain threshold, and thus vaccination would fail to fully protect from infection (47). We have attempted to enumerate the resting population of memory T cells in CpG + Inact-immunized mice 28 days after infection. While differences in CD44 expression were observed CD8 cells in the lung (Figure 6A), *ex vivo* restimulation and intracellular cytokine analysis revealed little difference between mock and CpG + Inact immunized groups. However, a trend of increased cytokine-positive cells combined with increases in CD44 expression in the lung suggests that CpG + Inact vaccination could be inducing resident memory T cells poised for effector function (48).

Conversely, the inability to detect the significant levels of cytokine-producing cells by peptide restimulation in CpG + Inact-immunized mice could be due to the low frequency of memory cells. The experiments here specifically look at responses to two peptides derived from M and NP each. Thus, the peptide restimulation might not be activating the full memory T cell repertoire, as memory responses could be generated to multiple conserved T cell epitopes (M, NP, PB1, PA) as well as non-conserved epitopes (H and N) present in the inactivated whole virus preparations. Furthermore, as the memory T cell population induced by vaccination may make up <0.5–2% of the total cells in the lung, isolating and staining these cells may be inefficient and not reflective of the true population. Nonetheless, the data suggest that memory responses are generated in CpG + Inact vaccinated mice as antigen-specific T cells are detected in the lungs 5 days post challenge (Figure 7).

Unexpectedly, the preparation of antigen played an important role in assessing the contribution of memory T cells to heterosubtypic protection. IAV can be inactivated in many different ways, each inducing unique immune responses. While heat-inactivated virus is less antigenic than other methods of viral inactivation (49), effector and memory T cell responses can clearly be generated when it is combined with CpG (Figures 5 and 7). Interestingly, very little influenza-specific class-switched IgG2a antibody was detected in the serum of immunized mice 4 weeks after immunization (Figures 6D,E), further supporting the pronounced role of memory T cells in heterosubtypic protection. One study found immunizing with a single dose of CpG and formalin-inactivated IAV increased anti-IAV antibodies 4 weeks post immunization in the serum and saliva (50). However, the modulation of the cellular immune response, measured by proliferation assays and CTL responses, was not observed. The difference between our study and the Moldoveanu report are likely due to antigen processing and presentation of the viral peptides.

Nonetheless, partial immune protection can serve as a framework to enhance and modify the existing platform to generate a vaccine with satisfactory efficacy and safety. For example, nanoparticle conjugation could be used for a dose sparing effect and enhanced protection. Other ways of modulating immunity could be through using multiple PRR ligands to generate a synergistic T cell response when combined with CpG. Our findings demonstrate a single dose of CpG, administered intranasally, can control viral replication and represents a possible strategy for developing vaccines against heterosubtypic infections in the future.

## Author Contributions

Conceived and designed the experiments: AV, DB. Performed the experiments: AV, DB. Data analysis and acquisition: AV. Drafted the work: AV. Critical revisions and final approval of the version to be published: DB.

## Conflict of Interest Statement

The authors declare that this research was conducted in the absence of any commercial or financial relationships that could be construed as a potential conflict of interest.

## Abbreviations

CTL, cytotoxic T lymphocyte; GrB, granzyme B; IAV, influenza A virus; IFN-γ, interferon gamma; PR8, influenza A/Puerto Rico/8/34; TNF-α, tumor necrosis factor alpha; x31, influenza A/Hong Kong/1/1968-X31.
